# Supplementary material for: Comparison of a Barcode-Based Smartphone Application to a Questionnaire to Assess the Use of Cleaning Products at Home and Their Association with Asthma Symptoms
Source: Int J Environ Res Public Health. 2021 Mar 24;18(7):3366. doi: 10.3390/ijerph18073366 (PMC8036634; doi:10.3390/ijerph18073366)

Comparison of a barcode-based smartphone application to a questionnaire to assess the use of cleaning products at home and its association with asthma symptoms

Pierre Lemire[1], Sofia Temam[1,2], Sarah Lyon-Caen[3], Catherine Quinot[1], Etienne Sévin[4], Sophie Remacle[1], Karine Supernant[3], Rémy Slama[3], Orianne Dumas[1], Valérie Siroux[3], Nicole Le Moual[1,] and the SEPAGES study group

[1] Université Paris-Saclay, UVSQ, Univ. Paris-Sud, Inserm, Équipe d'Épidémiologie Respiratoire Intégrative, CESP, 94807, Villejuif, France

[2] MGEN Foundation for Public Health (FESP-MGEN), 75748, Paris, France

[3] IAB, Team of Environmental Epidemiology Applied to Reproduction and Respiratory Health, INSERM U1209, University of Grenoble-Alpes, CHU de Grenoble – Grenoble, France

[4] Epiconcept, Paris, France

Supplementary Materials

Table S1: Cleaning products ingredients categorization; terms searched

**Bleach**	Hypochlorite de sodium<br>Hypochlorous acid<br>Sodium hypochlorite	Clorox<br>Antiformin<br>Javel
**Ammonia**	Ammoniaque<br>Ammoniac<br>Ammonia<br>Hydrogen nitride<br>Trihydrogen nitride<br>Sulfate d'ammonium	Ammonium sulfate<br>Nitrogen trihydride<br>Hydroxyde d'ammonium<br>Chlorure d'ammonium<br>Ammonium chloride
**Scented products**	Parfum<br>Parfums<br>Perfume<br>Perfumes<br>1-benzopyrane-2-one<br>2h-chromenone<br>Cumarine<br>Géraniol<br>Geraniol<br>(2e)-3,7-diméthylocta-2,6-dién-1-oltrans-3,7-diméthyl-2,6-octadién-1-ol<br>Isoeugenol<br>Salicyclate de benzyle<br>2-hydroxybenzoate de benzyle<br>O-hydroxybenzoate de benzyle<br>Citral<br>Pinus palustris oil<br>Limonene<br>D-limonene<br>Pinene<br>A-pinene	Lemonal<br>3,7-diméthyl-2,6-octadiènal<br>Geranial<br>Géranial<br>Neral<br>Néral<br>Nerol<br>Nerodol<br>Neraniol<br>Nérol<br>Nérodol<br>Cis-3,7-diméthyl-2,6-octadién-8-ol<br>(z)3,7-diméthyl-2,6-octadién-l-ol<br>Essential oil<br>Huile essentielle<br>Citronellol<br>3,7-diméthyloct-6-èn-1-ol<br>Linalol<br>Coumarine<br>Coumarines

Table S2: Cleaning products name scanned; text terms searched

**Bleach**	« javel » NOT « sans »	« bleach » NOT « without »
**Ammonia**	«ammonia»	«ammonia»
**Scented products**	«parfum»<br>«senteur»<br>«odeur»	«perfume»<br>«fragrance»<br>«odor»

Table S3: Included and excluded population characteristics at baseline in the SEPAGES study

	Included	Excluded	p(chisq)
**Questionnaire data**	101	324	
**Age (years)[a], mean ± s.d**	32.5 ± 3.6	32.5 ± 4.0	0.88
**Smoking status[b], n (%)**	101	324	
Current smoker	11 (10.9)	25 (7.7)	0.32
**Qualification level[a], n (%)**	88	295	0.52
Worker to supervisor or equivalent	41 (46.6)	149 (50.5)	
Manager or equivalent	47 (53.4)	146 (49.5)	
**Household Help, n(%)**	100	320	0.37
Participant alone	15 (15.0)	65 (20.3)	
Participant and help	76 (76.0)	220 (68.8)	
Help alone	9 (9.0)	35 (10.9)	
**Asthma Symptoms Score, n (%)**	96	316	
≥1	18 (18.8)	60 (19.0)	0.96
**Weekly spray use, n (%)**	91	305	
Yes	34 (37.4)	119 (39.0)	0.78
**Nb of weekly spray use, among users, n (%)**			
1	22 (24.2)	78 (25.6)	0.96
≥2	12 (13.2)	41 (13.4)	
**Weekly irritants use**	98	302	
Yes	45 (45.9)	142 (47.0)	0.85
**Nb of weekly irritants use, among users**	45	50	
1	39 (39.8)	110 (36.4)	0.41
≥2	6 (6.1)	32 (10.6)	
**Weekly bleach use, n (%)**	100	319	
Yes	10 (10.0)	37 (11.6)	0.66
**Weekly scented products use, n (%)**	98	307	
Yes	42 (42.9)	152 (49.5)	0.25
**Nb of weekly scented products use, among users n (%)**			
1	33 (33.7)	99 (32.2)	0.15
≥2	9 (9.2)	53 (17.3)	

[a] *before pregnancy*

[b] *between conception and pregnancy detection : non repeated data*

Table S4: Comparison of the two HDCP assessment methods at each of the three different data collection time

	Smartphone data<br>Weekly product use, n					
	**T1** [a]		**T3** [a]		**M2** [a]	
**Questionnaire data**<br>**Weekly spray use, n**	No	Yes	No	Yes	No	Yes
No	15	7	29	13	34	15
Yes	3	13	12	17	6	14
Concordance (%)/Discordance (%)	74/26		65/35		75/25	
Kappa coefficient [95%CI]	**0.47 [0.20-0.75]**		**0.27 [0.05-0.50]**		**0.35 [0.13-0.56]**	
Phi coefficient	**0.49**		**0.28**		**0.36**	
p [b]	**<0.01**		**0.03**		**<0.01**	
**Weekly bleach use, n**						
No	30	6	58	7	58	8
Yes	3	3	7	3	2	3
Concordance (%)/Discordance (%)	79/21		81/19		75/25	
Kappa coefficient [95%CI]	0.26 [-0.11-0.63]		0.19 [-0.11-0.49]		0.29 [-0.02-0.6]	
Phi coefficient	0.28		0.19		**0.34**	
p [b]	0.10		0.12		**0.02**	
**Weekly scented products use, n**						
No	1	18	9	30	9	39
Yes	1	20	2	31	1	20
Concordance (%)/Discordance (%)	53/47		56/44		42/58	
Kappa coefficient [95%CI]	0.0 [-0.15-0.16]		**0.16 [0.01-0.31]**		0.10 [-0.01-0.2]	
Phi coefficient	0.01		**0.24**		0.18	
p [b]	0.51		**0.05**		0.26	

[a] data collection times: first trimester of pregnancy(T1), third trimester of pregnancy(T3), second month after delivery(M2), [b] p for two-sided Fischer's exact test

Figure S1: Collection times for used exposure and respiratory data in the SEPAGES study

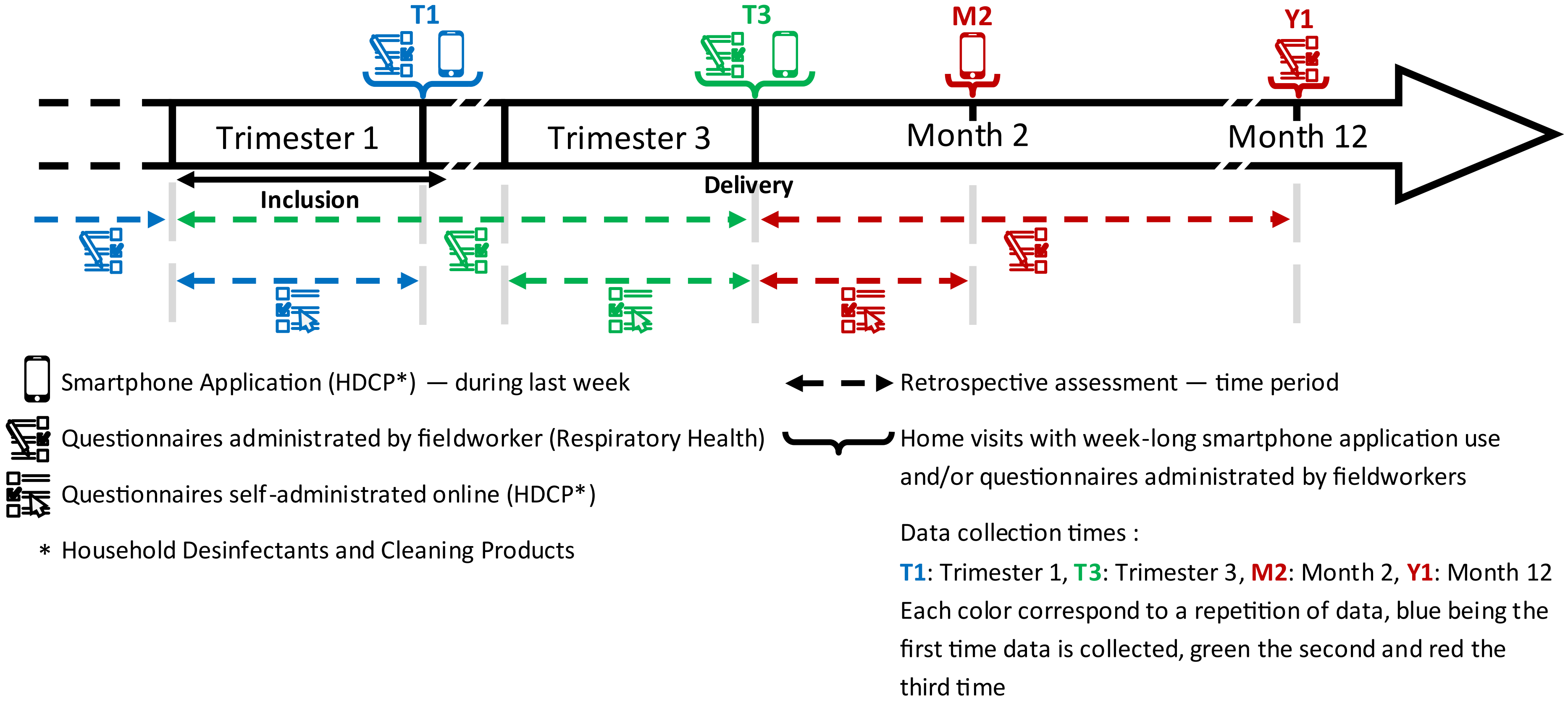

Supplement: Supplementary file 1 [file ijerph-18-03366-s001.pdf]
